# A case of V180I genetic mutation Creutzfeldt Jakob disease (CJD) with delusional misidentification as an initial symptom

**DOI:** 10.1080/19336896.2021.2017701

**Published:** 2021-12-29

**Authors:** Tomoyuki Nagata, Shunichiro Shinagawa, Nobuyuki Kobayashi, Kazuhiro Kondo, Masahiro Shigeta

**Affiliations:** a Department of Psychiatry, The Jikei University School of Medicine, Tokyo, Japan; b Department of Psychiatry, Airanomori Hospital, Kagoshima, Japan; c Department of Virology, The Jikei University School of Medicine, Tokyo, Japan

**Keywords:** Diffusion-weighted magnetic resonance image (DWI), neuropsychiatric symptom, creutzfeldt-jakob disease (CJD), V180i, delusional misidentification, prion protein gene (*PRNP*)

## Abstract

An 84-year-old woman who had been diagnosed as having dementia with Lewy body (DLB) upon initial examination exhibited cognitive impairments and person delusional misidentification (DMS): she transiently claimed that her spouse was a stranger. She was re-examined at the age of 89 years; her frequency of speech and activities of daily living had both decreased, leading to verbal communication difficulties complicated by sensory aphasia, and brain diffusion-weighted (DW) magnetic resonance imaging (MRI) showed cortical hyperintensities in some areas of both hemispheres. About 4 months later, the DW high-intensity areas were observed to have expanded into diffuse cortical areas. While the clinical features of Creutzfeldt Jakob disease (CJD) (myoclonus; ataxia; parkinsonism; rapidly progressive cognitive impairments; periodic sharp discharges on electroencephalograms) were not observed, a genetic analysis of the prion protein (*PRNP*) gene, which was performed because of a family history of dementia, revealed a V180I mutation (heterozygosis: valine/isoleucine) suggesting genetic CJD (g-CJD). Her activity progressively decreased, reaching akinetic mutism about 11 months after the re-examination. Finally, she suffered from severe bedsores and died from aspiration pneumonia at the age of 90 years. The present report describes the first case of person DMS as an initial neuropsychiatric symptom for V180I g-CJD; the typical long-term clinical symptoms of CJD were not observed in this patient. The inclusion of person DMS as an initial clinical symptom and the presence of expansive cortical hyperintensity areas may be useful for clinicians attempting to diagnosis V180I g-CJD in patients with elusive symptoms.

## Introduction

Prion diseases are neurodegenerative diseases caused by infectious abnormal-type prion deposition within cerebral tissue, leading to neuropathologically spongiform changes [1]. Prion diseases are classified into three types: (1) sporadic Creutzfeldt Jakob disease (s-CJD); (2) acquired infectious form including iatrogenic CJD or Kuru; and (3) genetic forms [1]. Prion protein gene (*PRNP*) mutations cause distinct clinical symptoms in patients with genetic forms, including Gerstmann-Sträussler-Scheinker disease (GSS), fatal familial insomnia (FFI), and genetic Creutzfeldt Jakob disease (g-CJD) [1]. In Japan, V180I (valine to isoleucine mutation at codon 180) is the most common mutation (approximately 40%) among g-CJD mutations and is associated with fewer typical clinical symptoms of CJD during the long-term dementia course [1,2]. Typical clinical symptoms of s-CJD include myoclonus, ataxia, parkinsonism, rapid cognitive impairments leading to dementia, a periodic sharp discharge (PSD) in electroencephalogram (EEG) findings, and elevated levels of 14-3-3 protein in cerebrospinal fluid (CSF) [1,2]. Therefore, concerning the initial clinical symptoms of V180I g-CJD, it may be difficult for clinicians to differentiate this condition from other dementia diseases [1,2]. Besides such representative clinical symptoms or surrogate markers differentiating g-CJD and s-CJD, cortical hyperintensities on diffusion-weighted magnetic resonance imaging (DW-MRI) have been shown to be a common finding [1,2]. The elusive clinical features of patients with the V180I mutation also show a lower penetrance; however, the course of the progressive dementia is known to finally reach akinetic mutism after some number of years [3].

Patients with dementia, including CJD, show various neuropsychiatric symptoms (NPSs) (delusion, hallucination, aggressiveness, apathy, and depression) from the initial stage until the terminal stage [4,5]. As NPSs in patients with CJD, personality change, appetite loss, sleep disturbance, emotional lability, psychosis, and depression have been reported [6–8]. On the other hand, in patients with V180I mutation among g-CJD, abnormal behaviours including pathological laughing, persecution delusion, wandering, and motivation loss have been shown as initial NPSs [9–11]. Among NPSs in patients with dementia, delusional misidentification syndrome (DMS) is a characteristic psychopathologic symptom in which ideations or feelings are consistently believed based on misinterpreted orientations (existence of persons, present places, or present situations); this symptom has been frequently documented in patients with dementia with Lewy body (DLB) or Alzheimer disease (AD) among neurodegenerative diseases [12,13]. The prevalence of DMS in both DLB and AD has each been reported to be about 16%. Capgras syndrome, Fregoli syndrome, and phantom border syndrome are representatives of distinctive DMS subtypes that concern the existence of a person (person DMS); in turn, person DMS is considered to differ from persecution delusion with regard to its symptomatic concept and psychopathological pathogenesis [13–15]. Reportedly, patients with person DMS can have harmful or dangerous behaviours arising from feelings of hostility towards objects or others; therefore, appropriate management or treatment for DMS may be necessary to prevent a worsening of caregiver burden [13,15]. To our knowledge, person DMS has been rarely reported as an atypical clinical symptom in patients with the V180I mutation. Therefore, a novel case report documenting the course of this DMS as an initial symptom may be important for clinicians considering a differential diagnosis in patients with DMS and progressive cortical hyperintensity areas as well as a family history of dementia, and such findings could be relevant to long-term treatment strategies.

The present case study was approved by the Ethics Committee of Airanomori Hospital (Kagoshima prefecture). To maintain patient privacy, all personal information has been altered in this case report. Moreover, written informed consent was obtained from both the patient and her main caregiver (her husband).

## Clinical summary

**Figure 1.**
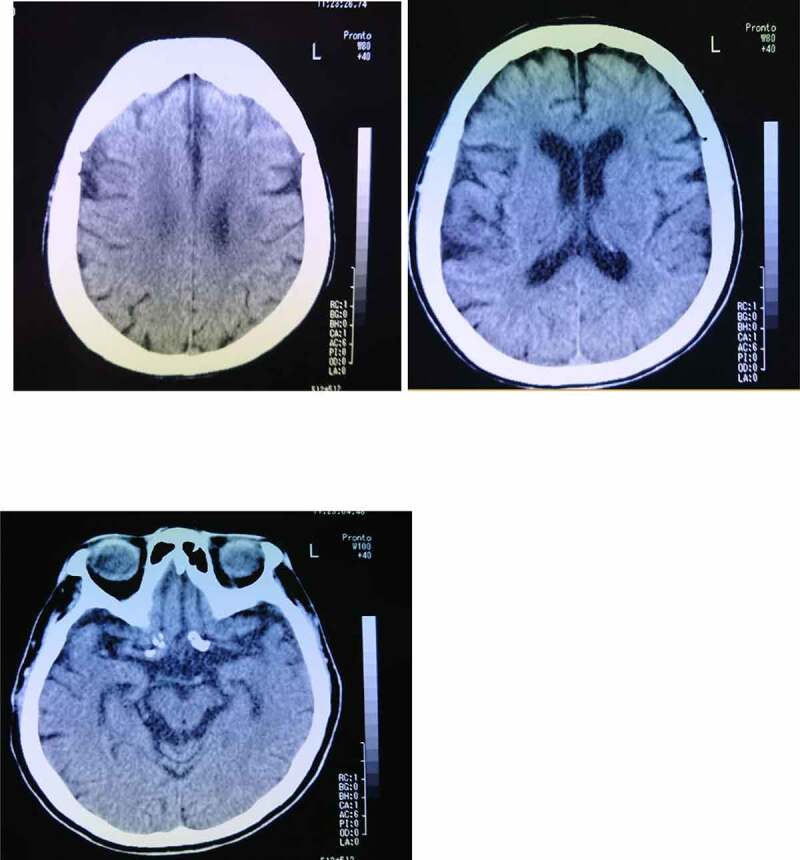
Brain computed tomography image of an 84-year-old woman shows mild cortical atrophy in the hippocampus and the absence of a cerebrovascular lesion.

An 83-year-old woman frequently claimed that her husband was a stranger or a newcomer, but her symptoms did not continue for long periods of time and typically disappeared in about 2–3 hours. She visited our outpatient memory clinic at the hospital with her husband to examine her cognitive impairment at the age of 84 years. She showed a mild cognitive impairment (memory impairment, disorientation of time, and lack of self-awareness about cognitive impairments), but no other neurological findings, including cranial nerve disorders, gait disturbances, weakness, sensory disturbances, ataxia, or aphasia, were present. Hypersomnia was observed in the evening as a diurnal rhythm disorder. Her right hand was dominant, and she had not experienced any significant problems with growth or development, substance abuse, or mental disorders and had no lifestyle diseases other than hypertension and hyperlipidaemia. Her siblings, including her brother and sister, had been diagnosed as having dementia, but she had neither a family history of CJD or associated iatrogenic exposure. Her Mini-Mental State Examination (MMSE) score was 18 points, and her self-care status corresponded to almost no need for assistance from other people. A brain computed tomography (CT) examination showed a mild cortical atrophy in the hippocampus and no remarkable cerebrovascular lesions (Figure 1). As NPS, both person DMS and a reduction in the frequency of outing activities, leading to disturbances in activities of daily living (ADL), were observed. The details of her person DMS were that she temporarily believed her husband to be ‘a stranger,’ despite having lived together for about 50 years, but her symptoms were transient. She could not identify her husband according to his voice, and she denied having person DMS when others corrected her. As a result, she was initially diagnosed at our hospital as having DLB. She was prescribed an anti-dementia medicine (donepezil) to prevent further cognitive decline, followed by outpatient examinations by her primary physician for about 5 years. She also began using a nursing-care service.

**Figure 2.**
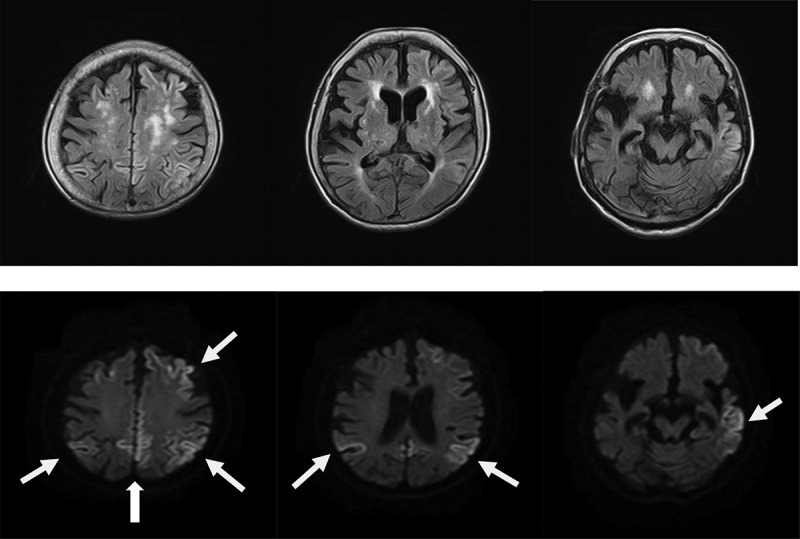
**b**. Magnetic resonance imaging (MRI) findings obtained using fluid-attenuated inversion-recovery (FLAIR) (upper) and diffusion-weighted images (DWI) (lower) in an 89-year-old woman at 4 months after the initial examination. DWI shows the expansion of the cortical hyperintensity areas in both hemispheres, relative to the previous findings.

**Figure 3.**
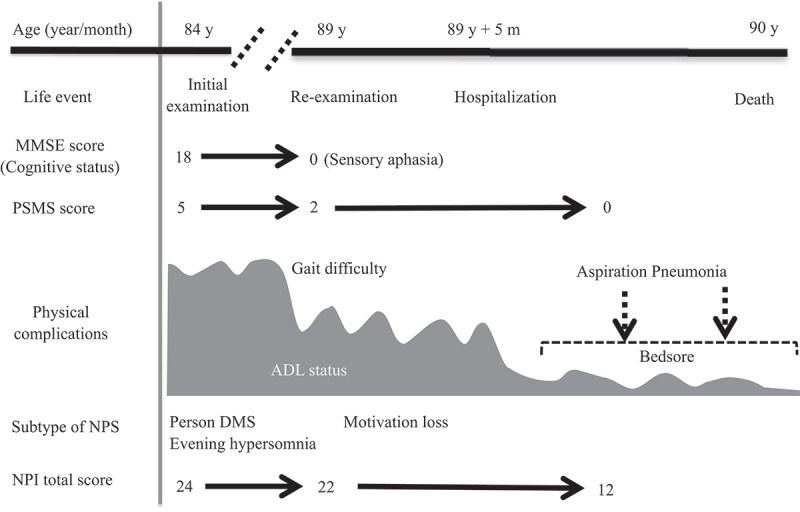
Clinical course of the present case with V180I CJD. In the present case, person DMS was first observed at the age of 84 years. The cognitive impairment progressed to motivation loss and sensory aphasia at the age of 89 years. The patient died because of aspiration pneumonia at the age of 90 years.

**Abbreviations**: ADL: activities of daily living, CJD: Creutzfeldt Jakob disease, DMS: delusional misidentification, MMSE: Mini-Mental State Examination, NPI: Neuropsychiatric Inventory, NPS: neuropsychiatric symptoms, PSMS: Physical Self- Maintenance Scale.

The patient was re-examined when she was 89 years old, and a depressive mood and labile affect were noted. Her speech frequency had also decreased, leading to verbal communication difficulties complicated by aphasia or mutism. Her MMSE score, reflecting her global cognitive status, was 0 points. She also had a poor self-care status (difficulty bathing, grooming, physical ambulation, and toilet excretion), which was thought to have been caused by a loss of motivation associated with her need for a wheelchair for locomotion. Other remarkable neurological findings (consciousness disturbances, hemiparesis, ataxia, involuntary movements including myoclonus, and abnormal pathological reflexes) were not observed. A brain DW-MRI examination showed cortical hyperintensities in some areas of both hemispheres (Figure 2a). Thus, she was thought to have multiple acute infarctions in cortical areas, and she was transferred to an emergency hospital. She received an intravenous drip treatment and rehabilitation against acute ischaemic stroke (anti-platelet, anti-oedema medicine, and neurological rehabilitation) and was discharged from hospital 8 days later. Four months after the re-examination at our hospital, she exhibited no altered neurological findings but a follow-up DW-MRI examination showed the expansion of the cortical hyperintensities with a ribbon pattern into both hemisphere (Figure 2b). Therefore, a more detailed laboratory and neurophysiological examination was performed in the department of neurology at another medical institution. An EEG examination did not show PSD, remarkable spikes, or slow waves; a CSF sample could not be obtained because of her inability to maintain a resting posture during sample collection. Blood laboratory data including the levels of thyroid hormone (free thyroxine), liver enzymes (aspartate transaminase, alanine transaminase, γ-glutamyl trans peptidase, alkaline phosphatase), an inflammatory marker (c-reactive protein), and blood glucose were within the normal ranges. To differentiate g-CJD from other dementia diseases based on the radiological findings of progressive cortical hyperintensity areas, the possible presence of a genomic DNA mutation was examined. As a result, a V180I mutation (heterozygosis: valine/isoleucine) of the *PRNP* gene was found; common polymorphisms were methionine homozygosity (M/M) at codon 129 and glutamate homozygosity (E/E) at codon 219. The genotype of *APOE* as a robust susceptible polymorphism gene was identified as homozygosity of a common variant (*ε*3/*ε*3). The general ADL of the patient had gradually decreased and had reached complete akinetic mutism without abnormal involuntary movements, resulting in severe bedsores. Finally, she died from aspiration pneumonia at the age of 90 years at about 13 months after the re-examination (Figure 3).
**Figure 2.**
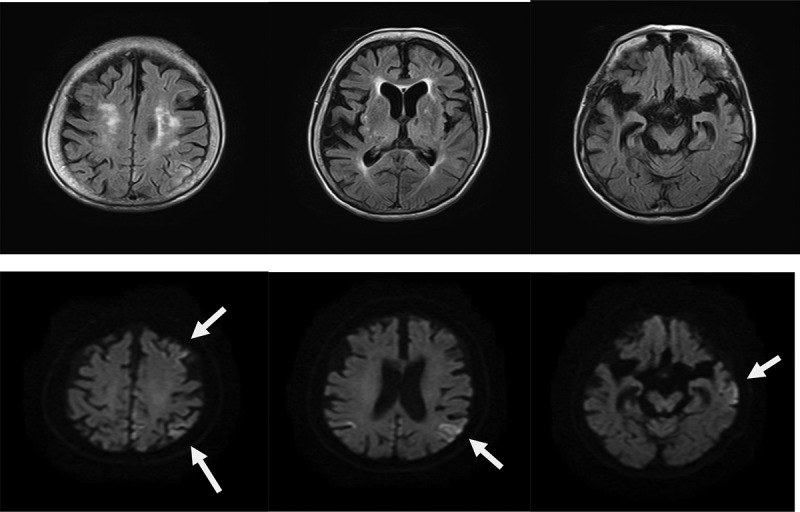
**a**. Magnetic resonance imaging (MRI) findings obtained using fluid-attenuated inversion-recovery (FLAIR) (upper) and diffusion-weighted images (DWI) (lower) in an 89-year-old woman. DWI shows cortical hyperintensities in some areas of both hemispheres; the white arrows indicate areas of cortical hyperintensity in the left hemisphere.

## Discussion

The clinical features of V180I CJD are as follows: (1) older onset age; (2) slower disease progression compared with sporadic CJD; (3) a relatively low occurrence rate of symptoms such as myoclonus, cerebellar symptoms, and visual disturbances; (4) a lower positive rate of brain-specific proteins, such as NSE, total tau protein, and 14-3-3 protein, in CSF; (5) a lack of PSD on EEG throughout the disease course; and (6) no family history of prion disease [3]. In the present case with dementia, the initial symptom during late-life onset was transient person DMS, similar to that in patients with DLB, but parkinsonism and visual hallucinations were not present [12,13,16]. However, the appearance of hypersomnia within a limited evening time might reflect a fluctuation in arousal, which is a core symptom of DLB [12,13,16]. These symptoms slowly progressed over a long period of 5 years and then gradually disappeared during the course of the dementia, and a hypoactive status or blunt affects appeared 6 years later. Moreover, the patient had a family history of dementia but with no clinical presentation of CJD. The clinical presentation of CJD in the present case was non-specific; however, the ribbon-like expansion of the hyperintense lesions in diffuse cortex suggested a diagnosis of CJD, and the identification of a genetic mutation confirmed the diagnosis and resolved what had been for many years a diagnostic mystery.

As initial NPSs in V180I CJD, distinct symptoms such as abnormal behaviours, pathological laughing, persecution delusion, wandering, and motivation loss have been reported (see Table 1) [9–11]. In one case with V180I CJD, a male patient with an onset at 78 years of age had typical theft delusion and moderate cognitive impairment as initial symptoms (similar to the clinical features of AD); this patient died 13 months after onset [9]. To our knowledge, one Japanese patient with V180I CJD showed parkinsonism and a neurocognitive disorder resembling a DLB clinical presentation with dopamine transporter single photon emission computed tomography (DAT-SPECT) showing reductions in tracer binding in the striatum 17, and previous reports have shown V180I cases to exhibit a senile plaque pathology in addition to CJD conventional pathological finding (spongiform changes and coexistence of pathological features of CJD and AD) [9,18–20]. Recently, the coexistence of a CJD, DLB, and AD pathology in one autopsy case with a conventional clinical presentation of CJD was reported [21]. Unfortunately, which causative pathology antecedently spread or whether the pathological overlap of AD, DLB and prion-related disease occurred remains unclear. However, the V180I mutation is located in the hydrophobic core of prion protein and may contribute to the conformational regulation (structural instability and misfolding) within the α helix structure via intramolecular interactions [22]. Especially, the alteration of valine to isoleucine at codon 180 may reduce the distance between the α2 to α3 helices and may regulate amyloid formation leading to an AD pathology and clinical presentation [22,23]. However, the genotype of *APOE* was identified as homozygosity of a common variant (*ε*3/*ε*3), and the susceptible gene might not have influenced the pathophysiology in the present case.

**Table 1.** Initial NPSs in cases with V180I genetic mutation CJD

Author (y)	Onset age (y)	Death age (y)	Sex	HIA in DW-MRI	PSD (EEG)	Family history	NPS as initial symptoms
Suzuki (2008) [9]	80	81	Male	Cerebral cortex and basal ganglia except for right occipital areas	Negative	None	Theft delusion Wandering
Iwasaki (2012) [10]	78	No data	Female	Cerebral cortex and basal ganglia except for medial occipital areas	Negative	None	Pathological laughing and crying
	76	No data	Female	Cerebral cortex and basal ganglia	Negative	None	Abnormal behaviours
	80	82	Male	No data	Negative	None	Pathological laughing and crying
Iwasaki (2019) [11]	87	No data	Female	Right hemisphere	Negative	None	Poor spontaneity
Left parietal and occipital lobes, except in the medial occipital regions							

**Abbreviation**: CJD: Creutzfeldt Jakob disease, DW-MRI: diffusion-weighted magnetic resonance imaging, EEG: electroencephalogram, HIA: hyperintensities, NPS: neuropsychiatric symptom, PSD: periodic sharp discharge

Some previous studies have investigated the anatomical neurobasis of DMS in patients with neurodegenerative diseases, including AD and DLB, and comprehensive reviews have noted a relation with the right frontal lobe and relevant surrounding regions [13,24]. The neurostructural alteration in patients with DMS and neurodegenerative diseases remains unclear, but neurofunctional changes have been observed in some regions or network domains within the same brain [13,24–27]. On the other hand, prosopagnosia appeared during a late phase in a previous case report of a patient with the V180I mutation, and mild atrophy in bilateral hippocampi was observed during the early phase [23]. While neurofunctional imaging was not performed in the present case, a brain CT examination performed at the age of 84 years showed mild cortical atrophy in the hippocampus (Figure 1). Whether person DMS as an initial symptom is related to mild hippocampal atrophy based on the coexistence of other neurodegenerative pathologies remains unclear; however, the misidentification of familiar faces might have occurred in the present case, similar to the above-mentioned patient [23]. DMS in patients with AD has been frequently reported and occurs during a moderately progressed stage of dementia (MMSE total score of about 13 points) [28]. In the present case, person DMS likely emerged during the initial stage of DLB because the symptoms did not have a long duration, like the fluctuations in the core symptoms of DLB [29]. However, AD-related pathologies contribute to the clinical symptoms of DLB from an early stage; thus, it may be difficult to clarify which pathological influence on the occurrence of person DMS was the most significant [30].

The present case was finally diagnosed as genetic CJD based on the confirmation of a genetic mutation, the appearance of distinct cortical ribbon-pattern hyperintensities on DW-MRI, and information regarding her family history of dementia. Structural neuroimaging, including DW-MRI, or hereditary information may be useful for diagnostic purposes; nevertheless, a lack of data (14-3-3 protein in CSF, SPECT or positron emission tomography) is a study limitation.

Despite a lack of definitive autopsy data, the clinical features in the present case were as follows: (1) person DMS as an initial symptom appeared during the progressive course of a patient with a V180I mutation in *PRNP*; (2) the slow progression was similar to the clinical presentation of DLB; and (3) the genetic mutation V180I provided a conclusive diagnosis of genetic CJD. Attention to unique initial symptoms and distinct radiological findings (cortical ribbon-pattern hyperintensities on DW-MRI) in patients with hereditary information may be helpful for clinicians to gain clues suggesting genetic analyses that could lead to long-term treatment strategies. Moreover, moving forward, further investigation of the relationship between person DMS and the V180I mutation in *PRNP* may help to elucidate the pathophysiology of DMS from genetic or molecular biological aspects.
